# Use of the Chinchilla Model to Evaluate the Vaccinogenic Potential of the *Moraxella catarrhalis* Filamentous Hemagglutinin-like Proteins MhaB1 and MhaB2

**DOI:** 10.1371/journal.pone.0067881

**Published:** 2013-07-02

**Authors:** Teresa L. Shaffer, Rachel Balder, Sean W. Buskirk, Robert J. Hogan, Eric R. Lafontaine

**Affiliations:** 1 Department of Microbiology, University of Georgia, Athens, Georgia, United States of America; 2 Department of Infectious Diseases, University of Georgia, Athens, Georgia, United States of America; 3 Department of Veterinary Biosciences and Diagnostic Imaging, University of Georgia, Athens, Georgia, United States of America; University of Kentucky College of Medicine, United States of America

## Abstract

*Moraxella catarrhalis* causes significant health problems, including 15–20% of otitis media cases in children and ∼10% of respiratory infections in adults with chronic obstructive pulmonary disease. The lack of an efficacious vaccine, the rapid emergence of antibiotic resistance in clinical isolates, and high carriage rates reported in children are cause for concern. In addition, the effectiveness of conjugate vaccines at reducing the incidence of otitis media caused by *Streptococcus pneumoniae* and nontypeable *Haemophilus influenzae* suggest that *M. catarrhalis* infections may become even more prevalent. Hence, *M. catarrhalis* is an important and emerging cause of infectious disease for which the development of a vaccine is highly desirable. Studying the pathogenesis of *M. catarrhalis* and the testing of vaccine candidates have both been hindered by the lack of an animal model that mimics human colonization and infection. To address this, we intranasally infected chinchilla with *M. catarrhalis* to investigate colonization and examine the efficacy of a protein-based vaccine. The data reveal that infected chinchillas produce antibodies against antigens known to be major targets of the immune response in humans, thus establishing immune parallels between chinchillas and humans during *M. catarrhalis* infection. Our data also demonstrate that a mutant lacking expression of the adherence proteins MhaB1 and MhaB2 is impaired in its ability to colonize the chinchilla nasopharynx, and that immunization with a polypeptide shared by MhaB1 and MhaB2 elicits antibodies interfering with colonization. These findings underscore the importance of adherence proteins in colonization and emphasize the relevance of the chinchilla model to study *M. catarrhalis*–host interactions.

## Introduction

*Moraxella catarrhalis* is a leading cause of otitis media worldwide along with *Streptococcus pneumoniae* and non-typeable *Haemophilus influenzae* (NTHi) [1], [2], [3], [4], [5], [6], [7], [8]. More than 80% of infants experience at least one episode of this disease by the age of three, and *M. catarrhalis* is the causative agent in ∼20% of these cases. Likewise, otitis media is the number one reason for which children are prescribed antibiotics [9], [10]. In the U.S., ∼25 million visits are made annually to pediatrician offices for treatment of this painful infection and of these, 3–5 million are precipitated by *M. catarrhalis* [1], [2], [3], [4], [5], [6], [7], [8], [11], [12], [13], [14], [15]. The annual costs associated with management of otitis media are upwards of $5 billion, and direct medical care expenditures alone account for $2–3 billion [1], [2], [5], [15], [16], [17], [18], [19]. The disease is a significant source of distress, as it produces a rapidly expanding middle ear abscess that exerts pressure on the tympanic membrane and causes acute stabbing pain. After the onset of otitis media, fluid persists in the middle ear for weeks to months and interferes with hearing. Recurring ear infections are prevalent and occur during the crucial period when a child is developing speech and language skills. Hence, many children spend most of the first 2–3 years of life with some hearing impairment because of multiple episodes of otitis media, which can delay the development of communication and learning. The WHO has estimated that chronic/recurrent otitis media occurs in 65–330 million people and is the major cause of hearing loss in developing countries [20], [21]. Clearly, otitis media is a significant health and economic problem, and *M. catarrhalis* contributes substantially to this burden.

*Moraxella catarrhalis* is also the second most common cause of respiratory infections in adults with chronic obstructive pulmonary disease (COPD) [19], [22], [23], [24]. This disease is the fourth leading cause of death in the U.S., surpassed only by heart attack, cancer and stroke [25]. Each year ∼10 million visits to physicians are related to COPD, and the costs associated with treatment are enormous – direct medical care costs alone are greater than $14 billion [26], [27], [28], [29]. Worldwide, COPD ranks as the fourth leading cause of death, killing more people than TB or HIV/AIDS, and is predicted to be third by 2030 [30], [31]. The course of this debilitating disease is characterized by intermittent exacerbations, half of which caused by bacterial infections. These infections, of which *M. catarrhalis* causes ∼10% of cases, contribute prominently to the progression of COPD by augmenting inflammation, oxidative stress, and tissue damage in the airways. In recent years, *M. catarrhalis* has also been increasingly associated with diseases such as bronchitis, conjunctivitis, and sinusitis [3], [6], [19], [32], [33], [34], [35], [36], [37], [38], [39], [40], [41], [42], [43], [44], [45], [46]. Long considered to be a harmless commensal of the respiratory tract, *M. catarrhalis* is now recognized as an important cause of infectious disease and a significant source of morbidity.

*M. catarrhalis* infections are a matter of concern due to the rapid emergence of antibiotic resistance in clinical isolates, high carriage rates in children, and the current lack of a vaccine. More than 90% of *M. catarrhalis* strains are now resistant to β-lactams [47], [48], [49], [50], [51], [52], [53], [54], which are generally the first antibiotics prescribed to treat otitis media. The genes specifying this resistance appear to be of Gram-positive origin [55], [56], suggesting that *M. catarrhalis* can readily acquire genes conferring resistance to additional antibiotics via horizontal transfer. Carriage rates as high as 81% have been reported in children [6], [57]. In one study, Faden and colleagues analyzed the nasopharynx of 120 children over a two-year period and showed that 77% of patients became colonized with *M. catarrhalis* [58]. These investigators also observed a direct relationship between colonization with *M. catarrhalis* and development of otitis media. This high carriage rate, coupled with the emergence of antibiotic resistance, suggests that *M. catarrhalis* infections may become more prevalent and difficult to treat, emphasizing the need to improve our understanding of pathogenesis by this understudied bacterium in order to identify targets for intervention and prevention.

To cause disease, *M. catarrhalis* must first colonize the nasopharynx and then spread to distal sites such as the middle ear and the lower respiratory tract. Hence, one key event that occurs early in pathogenesis by the organism is adherence to the mucosal surface of the nasopharynx because it leads to colonization. Crucial to this process are afimbrial adherence proteins (adhesins), which mediate binding of bacteria to host cells [59], [60], [61], [62], [63], [64], [65]. *Moraxella catarrhalis* expresses many afimbrial adhesins including UspA1 [66], UspA2H [66], MhaB1 and MhaB2 [67], MchA1 and MchA2 [68], Hag/MID [69], [70], OMPCD [71], [72], and McaP [73], [74]. These molecules were characterized by demonstrating a decrease in the adherence of mutant strains to human airway cells *in vitro,* but their contribution to nasopharyngeal colonization, or utility as vaccine antigens, has not been evaluated *in vivo*. In the present study, we utilized a chinchilla model to demonstrate that wild-type *M. catarrhalis* colonizes the nasopharynx for seven days, a mutant lacking expression of the adherence proteins MhaB1 and MhaB2 is impaired in its ability to colonize the nasopharynx, and immunization with a polypeptide shared by MhaB1 and MhaB2 elicits antibodies impeding nasopharyngeal colonization and promoting clearance.

## Materials and Methods

### Plasmids, Bacterial Strains, Growth Conditions, and Culture of Human Epithelial Cells *in vitro*

Strains and plasmids are described in Table 1. Wild-type (WT) *M. catarrhalis* isolates were routinely cultured using Todd-Hewitt agar plates (THA, BD Diagnostic Systems). The *M. catarrhalis* isogenic mutant strain O35E.B1B2 was propagated on THA supplemented with 15 µg/mL spectinomycin and 5 µg/mL zeocin. The *hag* transposon mutant O35E.TN2, the *ompCD* mutant strain O35E.CD1, and the *uspA2* serum-sensitive mutant O35E.2 were cultured using THA containing 20 µg/mL kanamycin. For colonization experiments, tissues and nasopharyngeal lavages collected from infected animals were plated onto THA supplemented with 5 µg/mL Vancomycin and 2.5 µg/mL Trimethoprim to suppress the growth of the chinchilla flora. *Escherichia coli* was grown using Luria-Bertani (LB) medium (Fisher BioReagents) containing 15 µg/mL chloramphenicol or 100 µg/mL ampicillin. All strains were cultured at 37°C in the presence of 7.5% CO_2_. The human cell line HEp-2 (laryngeal epithelium; ATCC CCL-23) was cultured as previously reported [74].

**Table 1:** Strains and plasmids used in this study.

Strain	Description	Source
***M. catarrhalis***		
O35E	WT isolate from middle ear effusion (Dallas, TX)	[82]
O35E.B1B2	*mhaB1mhaB2* double isogenic mutant of strain O35E,spectinomycin and zeocin resistant	[67]
O35E. TN2	*hag* transposon mutant of strain O35E, kanamycin resistant	[132]
O35E.2	*uspA2* isogenic mutant of strain O35E, kanamycin resistant	[133]
O35E.CD1	*ompCD* isogenic mutant of strain O35E, kanamycin resistant	[72]
O12E	WT isolate from middle ear effusion (Dallas, TX)	[66]
McGHS1	WT isolate from patient with respiratory infection (Toledo, OH)	[70]
***E. coli***		
EPI300™	Cloning strain for recombinant DNA methods	Epicentre® (Illumina®)
TUNER™	Expression strain for protein purification purposes	EMD Millipore
**Plasmids**		
pGEX4T-2	Protein expression vector, ampicillin resistant	GE Healthcare Life Sciences
pGEX-MhaB	pGEX4T-2 expressing O35E MhaB1 aa 72–399 joined to aGST N-terminal tag (GST-MhaB), ampicillin resistant	This study
pGEX-McaP	pGEX4T-2 expressing O35E McaP aa 51–333 joined to aGST N-terminal tag (GST-McaP), ampicillin resistant	This study
pRBHis.MhaB.72.399	pETcoco-1 expressing O12E MhaB1 aa 72–399 joined to6 N-terminal histidine residues (His-MhaB), chloramphenicol resistant	[67]

### Recombinant DNA Methods, PCR, and Cloning

Standard molecular biology techniques were performed as described elsewhere [70], [72], [74], [75]. Genomic DNA was obtained using the Easy-DNA™ kit (Invitrogen™ Life Technologies™). Platinum *Pfx* DNA Polymerase was used in cloning experiments per the manufacturer’s recommendations (Invitrogen™ Life Technologies™). A 1-kb amplicon encompassing amino acids (aa) 72–399 of the *M. catarrhalis* strain O35E MhaB1 protein was generated with primers P1 (5′-CGG GAT CCG TTA TTT CTG ACA GTC AAG CA- 3′; *BamHI* site underlined) and P2 (5′-CGC TCG AGT ATT ACC TTG CAA GTT GGC AGT- 3′; *XhoI* site underlined). This DNA fragment was excised from an agarose gel, purified with the High Pure PCR Product Purification Kit (Roche Applied Science), restricted with the endonucleases *BamHI* and *XhoI* (New England Biolabs® Inc.), and ligated into the *BamHI* and *XhoI* sites of the vector pGEX4T-2 (GE Healthcare Life Sciences), yielding plasmid pGEX-MhaB. This plasmid was sequenced to verify that no mutations were introduced during PCR and to confirm that the protein expressed from pGEX-MhaB corresponds to residue 72–399 of *M. catarrhalis* O35E MhaB1 fused to an N-terminal Glutathione-*S*-transferase (GST) tag. Plasmid DNA used as template in sequencing reactions was obtained with the QIAprep Spin Miniprep Kit (Qiagen). A similar approach was used to obtain the plasmid pGEX-McaP, which expresses residues 51–333 of *M. catarrhalis* O35E McaP joined to GST. The PCR product cloned into pGEX-McaP was amplified with primers P3 (5′-CGG GAT CCC AAG AAT TTA GCC AAA CCG TA-3′; *BamHI* site underlined) and P4 (5′-CGC TCG AGT CCC TGA AGG GTG AAT TTT ATC AGC -3′; *XhoI* site underlined). *M.* *catarrhalis* O35E genomic DNA was used as the template in all PCR-based cloning experiments.

### Nucleotide Sequence Analysis

Plasmids were sequenced at the University of Michigan sequencing core (http://seqcore.brcf.med.umich.edu/. Accessed 2013 Jun 4). Chromatograms were analyzed and assembled with the Sequencher software (Gene Codes Corporation). Sequence analysis was performed using Vector NTI (Invitrogen™ Life Technologies™).

### Protein Preparation

Outer membrane proteins were obtained from *M. catarrhalis* strains using the EDTA procedure of Murphy and Loeb [76]. The method used to prepare whole-cell lysates is described elsewhere [77], [78]. The His-tagged recombinant protein His-MhaB was obtained as previously outlined by Balder *et al* [67]. The plasmids pGEX-MhaB and pGEX-McaP were introduced in the *E. coli* strain TUNER™ (EMD Millipore) for the purpose of overexpressing and purifying the recombinant proteins GST-MhaB and GST-McaP, respectively. Expression was induced by adding isopropyl-β-d-thiogalactopyranoside (IPTG, final concentration of 1 mM) to broth cultures and incubating for 5 hours at 37°C with agitation (200-rpm). Bacteria were pelleted, followed by treatment with the BugBuster® HT protein extraction reagent (EMD Millipore) supplemented with rLysozyme™ (EMD Millipore) under the recommended conditions. Recombinant proteins were then purified using a GST Spin Purification Kit (Thermo Scientific Pierce) per the manufacturer's instructions. Protein concentrations were determined with a bicinchoninic acid (BCA) Protein assay kit (Thermo Scientific Pierce).

### Analysis of Selected Antigens

Equivalent protein amounts were resolved by sodium dodecyl sulfate-polyacrylamide gel electrophoresis (SDS-PAGE) and proteins were visualized by staining gels with the ProtoBlue™ Safe reagent (National Diagnostics). Alternatively, the resolved proteins were transferred to a polyvinylidene difluoride (PVDF) membrane (EMD Millipore) for western blot analysis. After transfer, the PVDF membranes were submersed in StartingBlock™ T20 (Thermo Scientific) and incubated for 1 hour at room temperature. The membranes were then probed overnight at 4°C with primary antibodies (Abs) diluted in StartingBlock™ T20. After this incubation, the membranes were washed with Phosphate-Buffered Saline (PBS) supplemented with 0.05% (vol/vol) Tween 20, followed by 1 hour incubation at room temperature with secondary Abs conjugated to Horse Radish Peroxidase (HRP) diluted in StartingBlock™ T20. After washing off the excess secondary Abs with PBS+0.05% Tween 20, protein bands were visualized by chemiluminescence using the Luminata™ Crescendo Western HRP substrate (EMD Millipore) and a Foto/Analyst Luminary/FX imaging system (Fotodyne Inc.).

For ELISA, duplicate wells of Immulon™ 2HB plates (Thermo Scientific Nunc) were coated overnight at 4°C with ∼1 µg of purified GST-MhaB protein. Excess unbound protein was removed by washing the wells with PBS+0.05% Tween 20, and the wells were then filled with PBS+0.05% Tween 20 containing 3% (wt/vol) dry milk and incubated for 1 hour at room temperature. After washing with PBS+0.05% Tween 20, the wells were probed overnight at 4°C with primary Abs diluted in PBS+0.05% Tween 20+3% dry milk. After this incubation, the wells were washed with PBS+0.05% Tween 20, followed by overnight incubation at 4°C with secondary Abs conjugated to HRP and diluted in PBS+0.05% Tween 20+3% dry milk. After washing off the excess secondary Abs with PBS+0.05% Tween 20, 100 µL of the SureBlue™ TMB Microwell Peroxidase Substrate (KPL) was added to wells. Color development indicative of antibody binding was measured by determining the absorbance of well contents at a wavelength of 650 nm using a µQuant™ Microplate Spectrophotometer (BioTek®). End-point titers were determined as described by Song *et al.* [79] and correspond to the highest fold dilution giving an optical density at 650 nm greater than the mean value plus 3 standard deviations of pre-immune samples.

### Antibodies

The murine monoclonal Abs 10F3 (specific for the *M. catarrhalis* iron transport protein CopB [80]), 5D2 (specific for the *M. catarrhalis* adhesin Hag [81]), 17H4 (specific for the *M. catarrhalis* serum resistance protein UspA2 [82]), and 1D3 (specific for the *M. catarrhalis* adhesin OMPCD [83]), His-tag® (EMD Millipore) and GST-Tag™ were used as primary Abs in western blot experiments in combination with goat anti-mouse HRP (IgG+IgA+IgM) secondary Abs (SouthernBiotech). For experiments using chinchilla samples as primary Abs (ELISA, Western blot), goat anti-rat Abs conjugated to HRP were utilized for detection. Goat anti-rat HRP (IgG) and HRP (IgG+IgA+IgM) were purchased from SouthernBiotech. Goat anti-rat HRP (IgA) Abs were obtained from Bethyl Laboratories, Inc.

### Adherence Assays

The WT *M. catarrhalis* strains O35E, O12E and McGHS1 were preincubated for 30 min at 37°C with samples (serum, nasopharyngeal lavage fluids) collected from naïve and vaccinated chinchillas. These bacteria were then used to perform adherence assays as previously described by Lipski and colleagues [73]. Briefly, bacteria were incubated for 30 min with HEp-2 human laryngeal cells seeded in 24-well tissue culture plates at a multiplicity of infection of 100 bacteria to 1 epithelial cell. The infected cells were then washed to remove unbound bacteria and treated with a solution containing saponin. Well contents were mixed, serially diluted, and spread onto agar plates to count colony-forming units (CFU). This value was used to calculate the number of inoculated bacteria that bound to HEp-2 cells. The adherence of *M. catarrhalis* preincubated with samples from control chinchillas (*i.e.* immunized with PBS) was set at 100%. The adherence of *M. catarrhalis* preincubated with samples from chinchillas vaccinated with the His-MhaB protein is presented as the percentage (± standard error) of that of *M. catarrhalis* preincubated with samples from control chinchillas. These assays were performed in triplicate in three or more separate experiments.

### Intranasal Inoculation of Chinchillas with *M. catarrhalis*

The method used to inoculate the nasopharynx of chinchillas was adapted from that described by Luke *et al.* [84], Bakaletz and colleagues [85], and more recently by Hoopman *et al* [86]. Healthy adult chinchillas (*Chinchilla lanigera*) were purchased from Rauscher’s Chinchilla Ranch (LaRue, Ohio). Prior to inoculation, the animals were anesthetized with by injecting ketamine (10 mg/kg, Fort Dodge®) and xylazine (2 mg/kg, Lloyd Laboratories) intramuscularly (i.m.). Once anesthetized, the animals were placed on their stomach. Using a 26 ½ gauge needle attached to 1 cc syringe, 0.2 mL of a *M. catarrhalis* suspension containing ∼1×10^9^ CFU was delivered intranasally (i.n.) by administering 5–10 µL droplets to alternating nasal openings and allowing droplets to be brought into the nasopharynx by the animal’s breathing. A total volume of 0.1 mL was administered per naris. *Moraxella catarrhalis* strains used to inoculate chinchillas were cultured on THA for 16–20 hr at 37°C. These plate-grown bacteria were suspended to a concentration of ∼5×10^9^ CFU/mL in PBS supplemented with 0.15% gelatin (PBSG) to maintain the viability of the organism. The *M. catarrhalis* suspension was also diluted and 100 µL aliquots were immediately spread onto THA supplemented with vancomycin and trimethoprim to determine the number of CFU inoculated into the nasal passages of the chinchillas. Back titration of inoculum was performed for all challenge experiments.

Viable *M. catarrhalis* was recovered from the nasopharynx of infected animals by performing nasopharyngeal lavages or by collecting and homogenizing nasopharyngeal tissues. Lavages were performed under anesthesia. Using a 1 cc syringe and a 26 1/2 gauge needle, 0.5 mL of PBSG was delivered at the entrance of one naris (in the form of 5–10 µL droplets) by passive inhalation and collected from the other naris (as it is exhaled) utilizing an needle-free 1 cc syringe. Portions of these lavages were serially diluted and plated onto THA supplemented with vancomycin and trimethoprim. After 24 hr incubation at 37°C, CFU were counted to determine the number of viable *M. catarrhalis* bacteria present in the fluids.

To harvest nasopharyngeal tissues, chinchillas were first anesthetized as described above. While under anesthesia, the animals were euthanized by delivering 1 mL of Beuthanasia®-D solution (Schering-Ploug Animal Health) via cardiac injection. This was accomplished by inserting 21 gauge, 1 ½ inch needle into the chest cavity beneath the xyphoid process and injecting the euthanasia solution directly into the heart. After assurance of death, decapitation was performed. Standard dissection techniques were used to remove the eyes, mandibles, and soft tissues covering the skulls. Following this, the heads were bisected along the nasal septum to expose the interior structures of the nasopharynx. The mucosa of the nasopharynx and of the ethmoid and nasal turbinates were collected, weighed and placed in 2 mL of PBSG. The nasopharyngeal tissues were then shredded, homogenized using a sterile glass dounce and pestle (Kimble Chase Life Science and Research Products), serially diluted, and plated onto selective media to determine the number of viable *M. catarrhalis* organisms.

### Immunization of Chinchillas

Serum and nasopharyngeal lavage fluids were collected from anesthetized chinchillas prior to immunization. Nasal fluids were collected as described above and stored at −80°C for later use. Blood was drawn by cardiac puncture. This was accomplished by inserting 21 gauge, 1 ½ inch needle into the chest cavity beneath the xyphoid process and removing blood directly from the heart. The samples were allowed to clot, centrifuged to remove red blood cells, and the sera were stored at −80°C. Blood samples and nasopharyngeal lavage fluids were also collected on days 19 and 44 post-immunization.

Vaccination was performed under anesthesia. Groups of chinchillas were immunized with PBS (control animals) or 80 µg of the His-MhaB protein. PBS and protein preparation were mixed with Complete Freund’s Adjuvant (CFA) in a 1∶1 ratio (vol/vol) and administered subcutaneously (s.c.). Booster vaccinations were performed on days 23 and 72. Animals were boosted with PBS or 80 µg of His-tagged protein mixed with Incomplete Freund ’s Adjuvant (IFA).

### Animal Research Ethic Statement

This study was carried out in strict accordance with the recommendations in the Guide for the Care and Use of Laboratory Animals of the National Institutes of Health. The protocol was approved by the Institutional Animal Care and Use Committee of the University of Georgia. All efforts were made to minimize suffering.

### Statistical Analyses

The paired *t* test was used to analyze data from adherence assays. *P* values <0.05 were found to be statistically significant. The results of nasopharyngeal colonization experiments were examined with the Wilcoxon signed rank test. All statistical analyses were performed using the Graph Pad Prism software.

## Results

### Use of the Chinchilla Model to Examine Colonization of the Nasopharynx by *M. catarrhalis*

**Figure 1:**
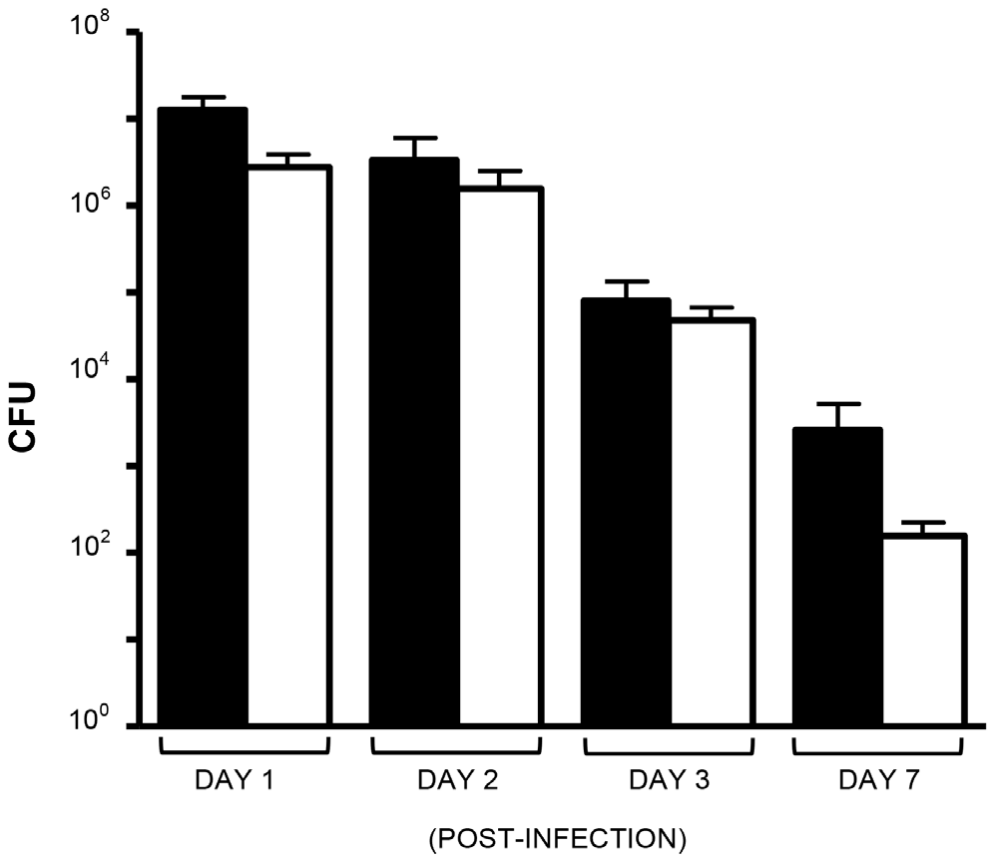
Recovery of WT *M. catarrhalis* O35E from the nasopharynx of chinchillas. Animals were inoculated with ∼1×10^9^ CFU. Results are expressed as the mean (± standard error) CFU/mL (lavage fluids, black bars) or CFU/gr (nasopharyngeal tissues, open bars). Each column represents at least 4 animals, and each experimental condition was tested on at least two separate occasions.

To study *M. catarrhalis* colonization and persistence *in vivo*, we developed the ability to utilize the chinchilla model of nasopharyngeal colonization. Figure 1 shows the results of calibration experiments in which chinchillas were inoculated intranasally (i.n.) with 10^9^ colony-forming units (CFU) of the wild-type (WT) isolate O35E. At the indicated times post-infection, animals were anesthetized and nasopharyngeal lavage fluids were collected, diluted and spread onto selective agar plates to suppress the growth of the chinchilla flora and accurately count viable *M. catarrhalis* CFU. Following this, chinchillas were euthanized and nasopharyngeal tissues were harvested, weighed, homogenized, diluted and plated. After overnight incubation at 37°C, CFU were counted to determine the number of viable *M. catarrhalis* bacteria present in lavage fluids and tissues. The results shown in Figure 1 demonstrates that we obtain reproducible and consistent numbers, comparable to those reported by Luke *et al.* for the WT isolate 7169 [84] and Hoopman and colleagues for strain O35E [86].

**Figure 2:**
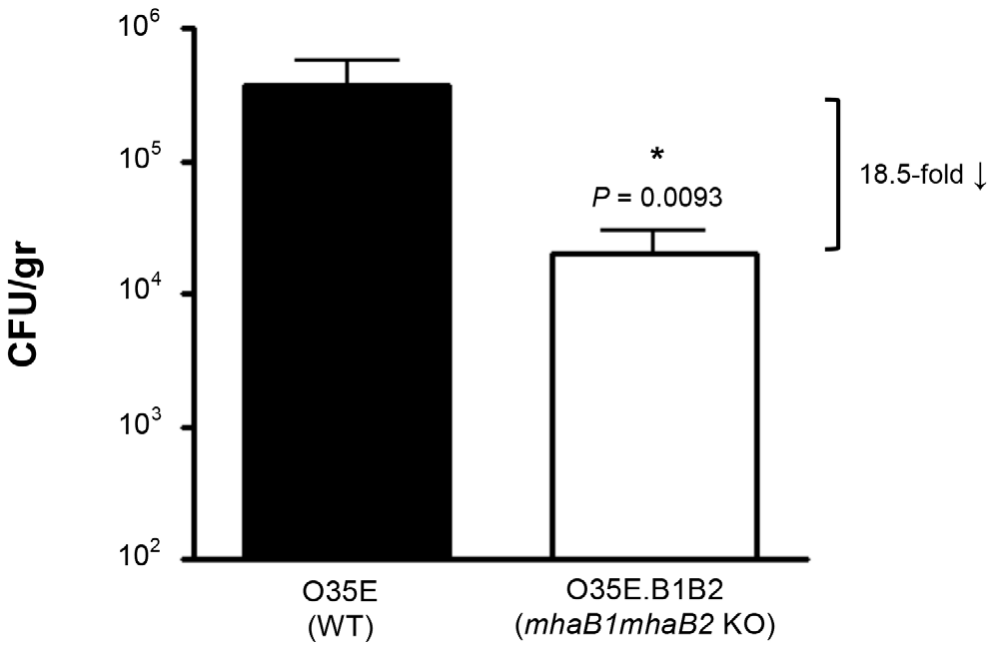
Recovery of *M. catarrhalis* from the nasopharynx of chinchillas three days post-infection. Animals were inoculated with ∼1×10^9^ CFU. Results are expressed as the mean (± standard error) CFU/gr of nasopharyngeal tissues. Strains were tested in parallel on two separate occasions. Each column represents 12 animals. The asterisk indicates that the reduction in the number of bacteria is statistically significant (Wilcoxon signed rank test).

After establishing the model, we tested the hypothesis that mutants lacking expression of adherence proteins will not colonize as effectively as WT *M. catarrhalis*. To accomplish this, cohorts of chinchillas were challenged with WT *M. catarrhalis* O35E and the mutant strain O35E.B1B2, which is unable to express the filamentous hemagglutinin-like proteins MhaB1 and MhaB2 [67]. These molecules are associated with the outer membrane of *M. catarrhalis* and are secreted in a Two-Partner Secretion manner via the outer membrane protein MhaC. MhaB1 and MhaB2 are involved in adherence to several human epithelial cell types that are relevant to the pathogenesis of *M. catarrhalis* (lung, laryngeal, conjunctival). The adhesins also resemble the filamentous hemagglutinin FHA, which is the major colonization factor of *Bordetella pertussis* and a component of all vaccines that are currently licensed for use in children to protect against whooping cough (CDC website. Available: http://www.cdc.gov/vaccines/pubs/pinkbook/downloads/pert.pdf. Accessed 2013 Jun 4). Figure 2 shows that lack of expression of MhaB1 and MhaB2 causes an 18.5-fold reduction in the number of viable *M. catarrhalis* bacteria recovered from nasopharyngeal tissues 72 hr post-infection. These results indicate that the filamentous hemagglutinin-like proteins are involved in *M. catarrhalis* ability to colonize and persist in the chinchilla nasopharynx. Lavages (prior to collecting tissues) were not performed in these experiments in order to generate a single value representing the total number of bacteria present in the nasal passageways at the experimental end-point.

### Use of the Chinchilla Model to Perform Vaccine Studies

**Figure 3:**
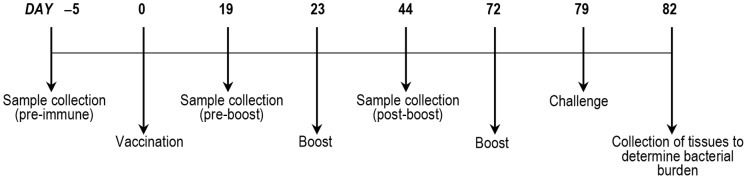
Timeline of vaccination experiments.

To test the hypothesis that a vaccine containing *M. catarrhalis* adherence proteins protects against colonization *in vivo*, chinchillas were immunized subcutaneously (s.c.) with a recombinant form of MhaB1 and MhaB2. Three independent vaccination trials were performed and the experimental timeline is depicted in Figure 3. The recombinant protein used to immunize chinchillas corresponds to aa 72–399 of MhaB1 fused to six N-terminal histidine residues. This portion of MhaB1 is 99% identical to aa 72–399 of MhaB2 in all *M. catarrhalis* isolates characterized to date, and murine Abs against this polypeptide were previously shown to react with both MhaB1 and MhaB2 [67]. This shared region of MhaB1 and MhaB2 also displays sequence similarity to the portion of *B. pertussis* FHA that is a component of all licensed vaccines for whooping cough (data not shown).

**Figure 4:**
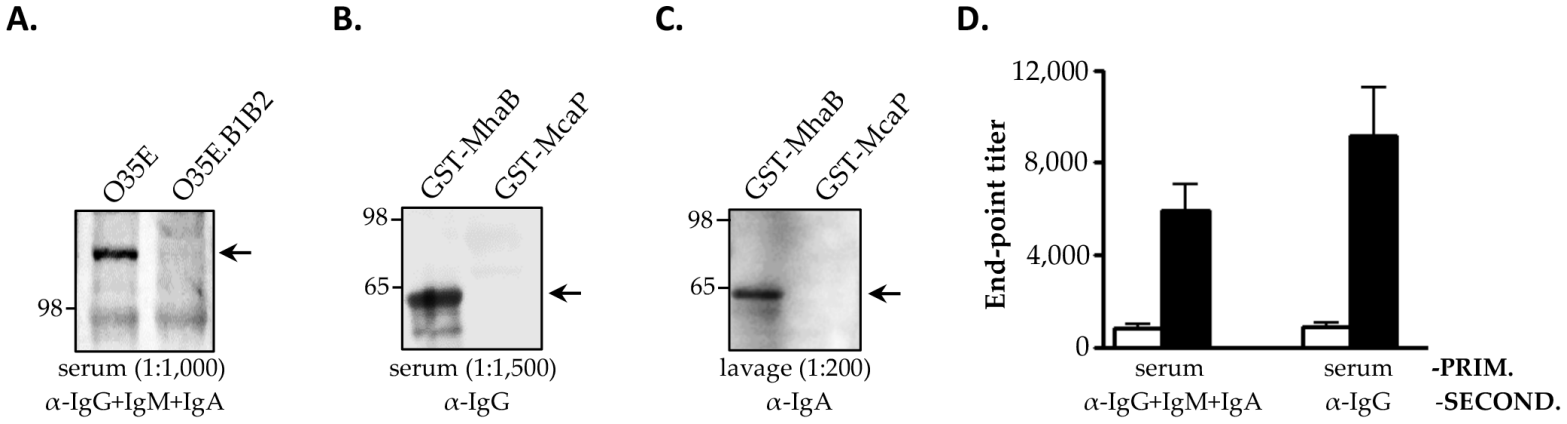
Western blot and ELISA analyses of samples from chinchillas immunized with the His-tagged MhaB protein. Western blot (panels A, B, C): Equivalent protein amounts were resolved by SDS-PAGE, transferred to PVDF and probed with the indicated primary and secondary Abs. Post-boost serum and lavage samples taken on Day 44 of the vaccination experiments (see Fig. 3) were pooled and used as primary Abs at the dilution shown in parentheses. Goat α-rat Abs conjugated to HRP were used as secondary Abs. Panel A: western blot of outer membrane protein preparations from the WT *M. catarrhalis* strain O35E and the *mhaB1mhaB2* mutant O35E.B1B2. Panels B and C: western blot of the purified recombinant proteins GST-tagged MhaB and GST-tagged McaP (used as negative control). Arrows indicate proteins specifically reacting with chinchilla Abs α-MhaB1/MhaB2. MW markers are shown to the left in kDa. ELISA (panel D): Individual serum samples were serially diluted and placed in duplicate wells of plates coated with GST-tagged MhaB. Goat α-rat Abs conjugated to HRP were used as secondary Abs. The results are expressed as the mean (± std deviation) end-point titer of samples from n = 12 animals. Individual titers were determined using pre-immune samples as background. Open bars correspond to pre-boost samples taken on Day 19 of the vaccination experiments while black bars represent post-boost samples collected on Day 44 (see Fig. 3).

**Figure 5:**
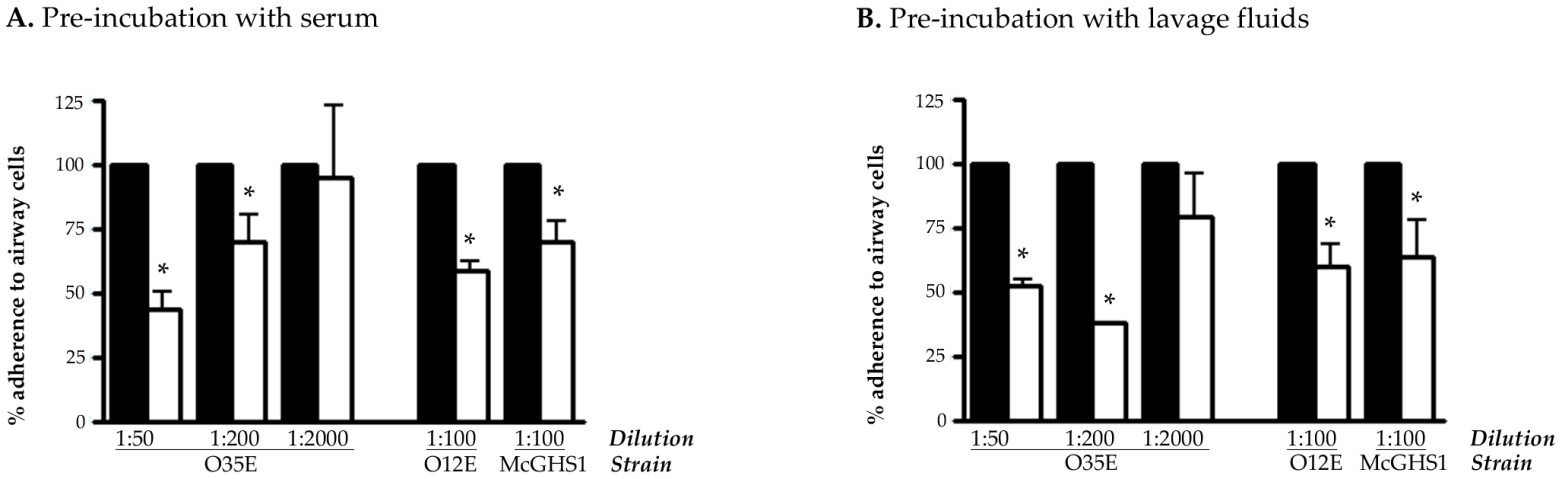
Inhibition of adherence with samples from chinchillas immunized with His-tagged MhaB protein. The WT *M. catarrhalis* strains O35E, O12E, and McGHS1 were preincubated for 30 min at 37°C with pooled samples from chinchillas sham-vaccinated with PBS (black bars) or with pooled samples from chinchillas immunized with His-tagged MhaB at dilutions of 1∶50, 1∶100, 1∶200 and/or 1∶2000. These bacteria were then used to perform adherence assays. The adherence of *M. catarrhalis* preincubated with samples from chinchillas vaccinated with PBS was set at 100%. The adherence of *M. catarrhalis* preincubated with samples from chinchillas immunized with His-tagged MhaB is expressed as the percentage (±standard error) of that of *M. catarrhalis* preincubated with samples from chinchillas vaccinated with PBS. Assays were performed in triplicate on three separate occasions. The asterisks indicate that the reduction in adherence is statistically significant (*P* values <0.05, paired *t* test). Post-boost samples taken on Day 44 of vaccination experiments (see Fig. 3) were pooled and used in these assays.

Serum and nasopharyngeal lavage fluids were collected from chinchillas and analyzed by western blot and ELISA. The results are shown in Figure 4 and demonstrate that the animals produced serum Abs reacting with the adhesins in the outer membrane of *M. catarrhalis* (Fig. 4A) and with a GST-tagged version of MhaB1/MhaB2 (Fig. 4B and 4D). The data also indicate that chinchillas developed mucosal Abs binding to the shared region of MhaB1 and MhaB2 (Fig. 4C). Serum and lavage fluids from the control animals vaccinated with PBS did not contain Abs against the adhesins (data not shown). Following this, we performed *in vitro* adherence assays in which *M. catarrhalis* was incubated with serum or lavage fluids from immunized chinchillas prior to infecting HEp-2 laryngeal cells. These experiments revealed that chinchilla Abs against MhaB1 and MhaB2 significantly decrease the adherence of multiple WT *M. catarrhalis* isolates to epithelial cells (Fig. 5A and 5B). The data also indicate that this inhibitory effect is dependent on the concentration of Abs.

**Figure 6:**
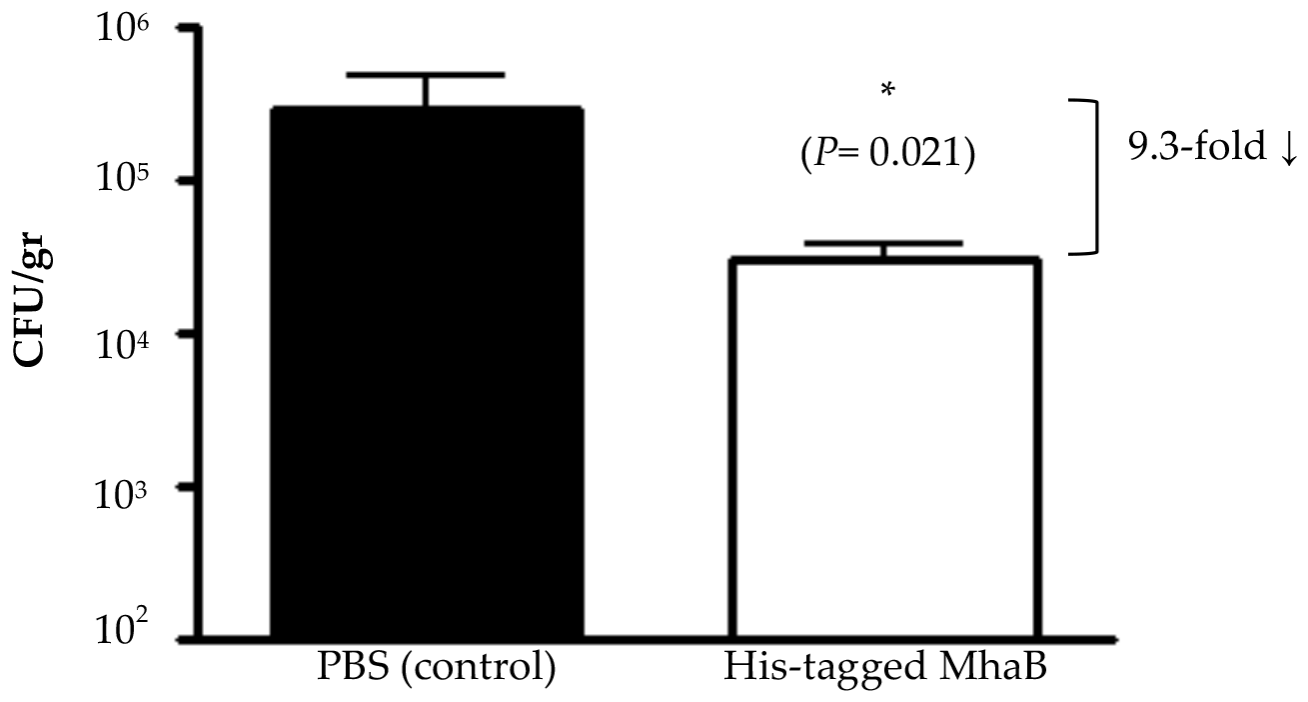
Recovery of WT *M. catarrhalis* O35E from the nasopharynx of immunized chinchillas three days post infection. Results are expressed as the mean (± std error) CFU/gr of nasopharyngeal tissues (note the log scale). The asterisk indicates that the reduction in the number of bacteria is statistically significant (Wilcoxon signed rank test, *P* value is shown in parentheses). Control and His-tagged MhaB groups were tested in parallel on three separate occasions. Each column represents 12 animals (groups of n = 4 animals/experiment).

After confirming that chinchillas produced Abs against MhaB1 and MhaB2, and demonstrating that these Abs interfere with adherence to airway cells, we challenged the animals with ∼10^9 ^CFU of the WT strain O35E and determined bacterial loads in nasopharyngeal tissues three days post-infection. Figure 6 shows that vaccination with the His-tagged MhaB protein causes a 9.3-fold reduction in the number of viable *M. catarrhalis* bacteria recovered from the nasopharynx of chinchillas compared to sham-immunized animals. These results substantiate the data obtained when comparing the ability of the mutant O35E.B1B2 to colonize the nasopharynx to that of its progenitor strain O35E (Fig. 2). The results also support the hypothesis that a vaccine containing *M. catarrhalis* adherence proteins will elicit the production of Abs blocking colonization and promoting clearance.

### *Moraxella catarrhalis* Proteins Targeted by the Chinchilla Immune Response During Colonization

**Figure 7:**
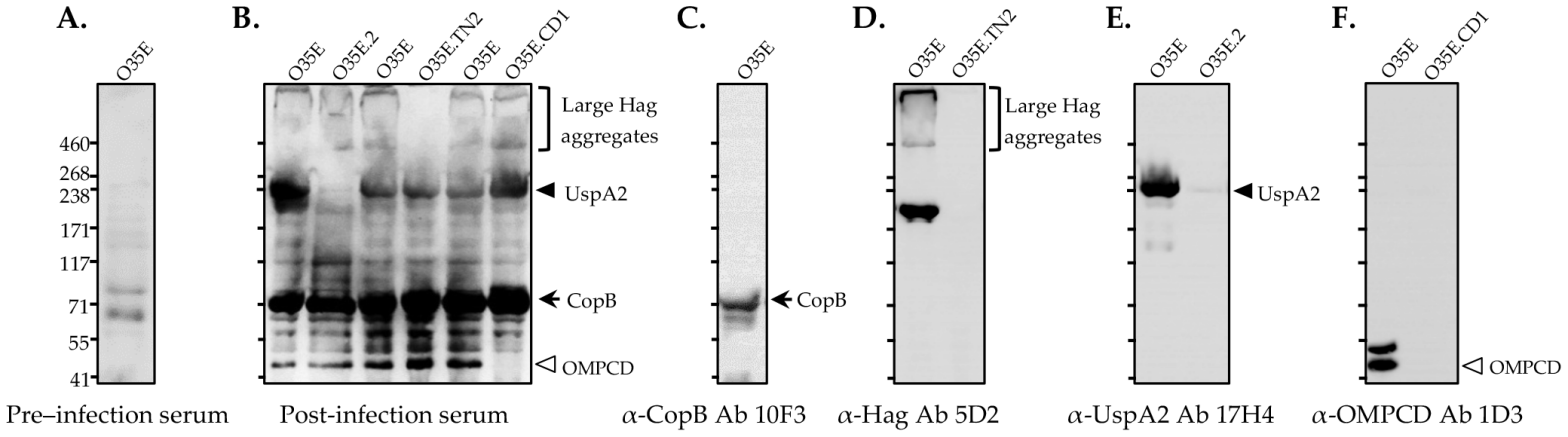
Western blot analysis of serum from chinchillas inoculated with the WT *M. catarrhalis* strain O35E. Equivalent amounts of whole cell lysates (WT *M. catarrhalis* O35E, *uspA2* KO strain O35E.2, *hag* transposon mutant strain O35E.TN2, and *ompCD* KO strain O35E.CD1) were resolved by SDS-PAGE, transferred to PVDF and probed with the indicated primary Abs. Panels A and B: Pre- and post-infection serum samples were pooled and used as primary Abs at a dilution of 1∶250. Goat α-rat IgG conjugated to HRP were used as secondary Abs. Controls: The murine monoclonal Abs 10F3 (Panel C, α-CopB), 5D2 (Panel D, α-Hag), 17H4 (Panel E, α-UspA2) and 1D3 (Panel F, α-OMPCD) were used as primary Abs in combination with goat α-mouse HRP-(IgG+IgA+IgM) secondary Abs. These controls were included to verify the identity of proteins recognized by post-infection chinchilla serum in panel B. MW markers are shown to the left of in kDa.

To gain more insight into the immune response of the chinchilla to *M. catarrhalis*, we inoculated four animals i.n. with 10^9^ CFU of the WT strain O35E on three consecutive occasions (21 days apart). Seven days after the last challenge, serum samples were collected and analyzed by western blot. Figure 7 shows that chinchillas produced Abs against several *M. catarrhalis* antigens during colonization including the iron acquisition protein CopB, the serum-resistance factor UspA2, and the adhesins OMPCD and Hag. Of significance, these four molecules have been shown to be major targets of systemic and mucosal antibody responses in humans [83], [87], [88], [89], [90], [91], [92], [93], [94]. Infected chinchillas did not produce detectable levels of Abs against the shared region of MhaB1 and MhaB2 (data not shown).

## Discussion

The success of the immunization program against *S. pneumoniae* has placed more emphasis on *M. catarrhalis* as a frequent cause of ear infection. Vaccination of children with Prevnar®, which contains capsular polysaccharides from seven different *S. pneumoniae* serotype strains, affords protection against otitis media caused by the organism (57% efficacy) [95]. Likewise, an investigational vaccine containing the capsule of 11 distinct *S. pneumoniae* serotype strains conjugated to protein D of *H. influenza* was shown to reduce the incidence of ear infection caused by *S. pneumoniae* (57% efficacy) and NTHi (35% efficacy) [96]. Significantly, Synflorix™, a capsule-protein D conjugate vaccine comprising capsular polysaccharides from 10 different *S. pneumoniae* serotype strains, was licensed in Europe in 2009. While these studies demonstrate that prevention of otitis media can be achieved, the widespread administration of capsule-protein D conjugate vaccines protecting against both *S. pneumoniae* and NTHi, along with the continued expansion of the *S. pneumoniae* vaccination program (a version of Prevnar® covering 13 capsule serotypes was licensed in 2010), will result in *M. catarrhalis* becoming an even more prevalent cause of infectious disease. Evidence of such a shift has been observed in children with otitis media as well as in children and adults with sinusitis [97], [98], [99]. Therefore, the prevention of *M. catarrhalis* infections would make a significant contribution to improving children’s health. Though otitis media would be the primary target, a vaccine against the organism would also be of value to adults at high risk of infection, especially those with COPD.

*Moraxella catarrhalis* is an exclusively human organism and studying pathogenesis, as well as the stringent testing of vaccine candidates, has been hindered by the lack of an animal model that mimics human infection. To date, the most commonly used model has been a pulmonary clearance test in which bacteria are deposited in the lungs of mice [100], [101], [102], [103], [104], [105], [106]. Viable organisms are enumerated by aseptically removing the lungs, homogenizing the tissues, and spreading dilutions of the homogenates onto agar plates. While this model has provided important data, it is limited to measuring the rate at which bacteria are cleared over a very short period of time because *M. catarrhalis* persists for <24-hr in the murine lungs. Another drawback is that mice do not develop pneumonia. Hence, the rapid clearance and failure to cause disease limit the usefulness of this model.

Recent studies have demonstrated the value of the chinchilla to examine *M. catarrhalis* host-pathogen interactions *in vivo* [84], [85], [86], [107], [108]. Following intranasal inoculation, *M. catarrhalis* causes symptoms of disease (inflammation of the tympanic membrane, middle ear effusions) and colonizes the nasopharynx for ∼2 weeks [85], [86], [108]. Therefore, chinchillas provide an advantage over the mouse pulmonary clearance test in that *M. catarrhalis* persists in their nasopharynx for several days. This imparts greater confidence in the data obtained by comparing the difference in colonization between two experimental conditions (vaccinated vs. sham-vaccinated animals, WT vs. mutant strains) as it provides a more physiologically relevant time frame to monitor bacterial clearance. The chinchilla model has been an invaluable tool to study the pathogenesis of NTHi and *S. pneumoniae* and to develop vaccines for these organisms [109], [110], [111], [112]. The course of disease (nasopharyngeal colonization, ascension of the Eustachian tubes, development of middle ear effusions, clearance of fluids, return to homeostasis) is similar to that in children with otitis media [113], [114], [115], [116], [117], [118]. Immunological parallels between chinchillas and humans have also been demonstrated. For example, middle ear fluids collected from chinchillas and children infected with NTHi contain Abs that bind to the same antigenic determinants of the adhesin OMP P5 [119]. Chinchillas also produce homologs of human antimicrobial peptides, and at least 2 of them (cBD-1 and cCRAMP) have been shown to have bactericidal activity against *M. catarrhalis* [120], [121], [122], [123]. Kerschner and colleagues analyzed host cDNA libraries generated from the middle ear mucosa of chinchillas infected with NTHi, and discovered that the cDNA sequences displayed greater phylogenetic similarities to human genes than to other rodent species [124], [125], [126]. These investigators also noted similarities with human infection in the pattern of host defense genes expressed in chinchilla tissues. Our data showing that chinchillas infected with *M. catarrhalis* produce Abs against antigens known to be major targets of the immune response in humans further underscore the usefulness of the model (Fig. 7). To our knowledge, this is the first demonstration of immunological parallels between chinchillas and humans during *M. catarrhalis* infection.

We discovered that lack of expression of the filamentous hemagglutinin-like proteins MhaB1 and MhaB2 decreases recovery of viable *M. catarrhalis* cells from the chinchilla nasopharynx approximately 20-fold (Fig. 2). This reduction is most likely caused by a defect in adherence to the airway mucosa. MhaB1 and MhaB2 mediate adherence to respiratory cells *in vitro* and resemble FHA, the major adhesin and colonization factor of *B.* *pertussis* [67]. Moreover, Abs against MhaB1 and MhaB2 reduce *in vitro* adherence of *M. catarrhalis* (Fig. 5) and decrease the number of viable organisms recovered from the nasopharynx of chinchillas infected with the WT strain O35E (Fig. 6). Taken together, our data suggest that MhaB1 and MhaB2 are critical factors for colonization. Hoopman and colleagues recently used the chinchilla and DNA microarray technology to determine global transcriptome expression by *M. catarrhalis in vivo* [86]. More than 100 ORFs of strain O35E, including *mhaB1*, were found to be upregulated 24-hr after introducing the organism in the nasopharynx. Another 200 genes were shown to be downregulated, and the ORF specifying MhaB2 (MchA1) exhibited some of the highest levels of repression. Therefore, it is tempting to speculate that lack of MhaB1 is responsible for the reduced number of viable O35E.B1B2 cells recovered from the chinchilla nasopharynx during our colonization experiments (Fig. 2). However, the contribution of MhaB2 cannot be ruled out. The transcriptome analysis showing decreased *mhaB2* expression levels was performed with samples collected 24-hr post-inoculation, whereas we calculated bacterial loads in the nasopharynx 3 days after infection. It is possible that expression of *mhaB2* (and *mhaB1*) changes during this 48-hr period. Interestingly, microarray data also indicate that expression of the *uspA2* and *hag* genes is downregulated [86]. The western blot results of Fig. 7 show that infected chinchillas produce Abs against UspA2 and Hag, demonstrating their expression *in vivo*. Clearly, understanding the individual contribution of MhaB1 and MhaB2 to colonization and persistence is a key area for future study.

Although lack of MhaB1 and MhaB2 reduces the recovery of viable O35E.B1B2 cells from the chinchilla nasopharynx, the mutant retained colonization capabilities (Fig. 2), which implies that additional factors contribute to this process. Luke *et al* used the chinchilla model to show that a type IV pilus mutant of *M. catarrhalis* strain 7169 does not colonize as effectively as the WT parent isolate [84]. The pilus-negative mutant exhibited 7.67-, 2.56-, and 9.6-fold reductions in recovery of viable organisms from nasopharyngeal, nasoturbinate, and ethmoid turbinate tissues, respectively. The mutant also showed lower adherence to epithelial cells *in vitro* [84]. Strain O35E expresses a type IV pilus [127], which presumably contributed to colonization in our experiments. Our laboratory demonstrated that *M. catarrhalis* has strict tropism for ciliated cells of the human respiratory tract and that the autotransporter adhesin Hag is responsible for this phenotypic trait [69]. Brockson and colleagues recently reported that *M. catarrhalis* exhibits similar ciliotropism in the chinchilla nasal passageways [108]. Hag may therefore play a role in colonization and persistence. Other potential colonization factors include UspA1 (binds to human CEACAM-1 receptor [128], [129], [130], chinchillas express a homologue of human CEACAM-1 shown to be necessary for colonization by NTHi [131]) and genes that are part of the truncated denitrification regulon, specifically MC ORF1550 (encodes a protein of unknown function, highly upregulated in the chinchilla nasopharynx 24-hr post inoculation, mutation in the gene causes a decrease in the ability of strain O35E to survive in the chinchilla nasopharynx over a 3-day period [86]).

The results of vaccination experiments validate the role of MhaB1 and MhaB2 as critical factors for colonization. Subcutaneous immunization with a polypeptide common to both molecules elicits the production of serum Abs reacting with the proteins in the outer membrane of *M. catarrhalis* (Fig. 5A). Vaccinated animals also develop mucosal Abs binding to the shared region of MhaB1 and MhaB2 (Fig. 5C). These Abs not only block *M. catarrhalis* adherence *in vitro*, but also reduce nasopharyngeal colonization of the WT strain O35E by one order of magnitude (Fig. 6). The MhaB proteins function as adhesins and mediate a key step in pathogenesis by *M. catarrhalis*. To cause disease, the organism must first colonize the nasopharynx and then spread to distal sites such as the middle ear and the lower respiratory tract. Hence, adherence to the mucosal surface of the nasopharynx is critical. MhaB1 and MhaB2 are surface-located and thus are readily accessible to Abs and the host immune response. In addition, the proteins are well conserved among clinical isolates of diverse clinical and geographical origins [67], [68]. Therefore, MhaB1 and MhaB2 possess many attributes of excellent vaccine candidates. Our results showing that Abs against the shared region of MhaB1 and MhaB2 blocks adherence of multiple WT *M. catarrhalis* isolates suggests that immunization with the proteins will have broad-spectrum activity. Of note, this shared region of MhaB1 and MhaB2 displays sequence similarity to the portion of *B. pertussis* FHA that is a component of all vaccines that are currently licensed for use in children to protect against whooping cough. Future studies will be aimed at exploring the vaccinogenic potential of MhaB1 and MhaB2 with adjuvants that readily translate to human studies, immunization routes that promote robust mucosal immunity, measuring colonization at multiple intervals post-inoculation, and testing additional *M.* *catarrhalis* isolates.

## References

[1] Cripps AW, Otczyk DC, Kyd JM (2005) Bacterial otitis media: a vaccine preventable disease? Vaccine 23: 2304–2310. 15755616 10.1016/j.vaccine.2005.01.023

[2] Giebink GS, Kurono Y, Bakaletz LO, Kyd JM, Barenkamp SJ, et al.. (2005) Recent advances in otitis media. 6. Vaccine. Ann Otol Rhinol Laryngol Suppl 194: 86–103. 15700938

[3] Karalus R, Campagnari A (2000) *Moraxella catarrhalis*: a review of an important human mucosal pathogen. Microbes Infect 2: 547–559. 10865200 10.1016/s1286-4579(00)00314-2

[4] Murphy TF (2005) Vaccine development for non-typeable Haemophilus influenzae and Moraxella catarrhalis: progress and challenges. Expert Rev Vaccines 4: 843–853. 16372880 10.1586/14760584.4.6.843

[5] Pichichero ME, Casey JR (2002) Otitis media. Expert Opin Pharmacother 3: 1073–1090. 12150687 10.1517/14656566.3.8.1073

[6] Verduin CM, Hol C, Fleer A, van Dijk H, van Belkum A (2002) Moraxella catarrhalis: from emerging to established pathogen. Clin Microbiol Rev 15: 125–144. 11781271 10.1128/CMR.15.1.125-144.2002 PMC118065

[7] Faden H (2001) The microbiologic and immunologic basis for recurrent otitis media in children. Eur J Pediatr 160: 407–413. 11475577 10.1007/s004310100754

[8] Enright MC, McKenzie H (1997) Moraxella (Branhamella) catarrhalis–clinical and molecular aspects of a rediscovered pathogen. J Med Microbiol 46: 360–371. 9152030 10.1099/00222615-46-5-360

[9] Arguedas A, Kvaerner K, Liese J, Schilder AG, Pelton SI (2010) Otitis media across nine countries: disease burden and management. Int J Pediatr Otorhinolaryngol 74: 1419–1424. 20965578 10.1016/j.ijporl.2010.09.022

[10] Diagnosis and management of acute otitis media. Pediatrics 113: 1451–1465. 10.1542/peds.113.5.1451 15121972

[11] Del Beccaro MA, Mendelman PM, Inglis AF, Richardson MA, Duncan NO, et al (1992) Bacteriology of acute otitis media: a new perspective. J Pediatr 120: 81–84. 1731029 10.1016/s0022-3476(05)80605-5

[12] Faden H, Duffy L, Wasielewski R, Wolf J, Krystofik D, et al (1997) Relationship between nasopharyngeal colonization and the development of otitis media in children. Tonawanda/Williamsville Pediatrics. J Infect Dis 175: 1440–1445. 9180184 10.1086/516477

[13] Faden H, Stanievich J, Brodsky L, Bernstein J, Ogra PL (1990) Changes in nasopharyngeal flora during otitis media of childhood. Pediatr Infect Dis J 9: 623–626. 2122410

[14] Ruuskanen O, Heikkinen T (1994) Otitis media: etiology and diagnosis. Pediatr Infect Dis J 13: S23–S26; discussion S50–S54. 8159511

[15] Stool SE, Field MJ (1989) The impact of otitis media. Pediatr Infect Dis J 8: S11–S14. 2494641

[16] Klein JO (1994) Otitis media. Clin Infect Dis 19: 823–833. 7893865 10.1093/clinids/19.5.823

[17] Klein JO (2000) The burden of otitis media. Vaccine 19 Suppl 1S2–8. 11163456 10.1016/s0264-410x(00)00271-1

[18] Klein JO, Teele DW, Pelton SI (1992) New concepts in otitis media: results of investigations of the Greater Boston Otitis Media Study Group. Adv Pediatr 39: 127–156. 1442312

[19] Murphy TF (1996) *Branhamella catarrhalis*: epidemiology, surface antigenic structure, and immune response. Microbiol Rev 60: 267–279. 8801433 10.1128/mr.60.2.267-279.1996 PMC239443

[20] Acuin J (2004) Chronic suppurative otitis media: burden of illness and management options. World Health Organization website. Available: http://www.who.int/pbd/deafness/activities/hearing_care/otitis_media.pdf). Accessed 2013 June 4.

[21] Berman S (1995) Otitis media in developing countries. Pediatrics 96: 126–131. 7596700

[22] Murphy TF, Brauer AL, Grant BJ, Sethi S (2005) Moraxella catarrhalis in chronic obstructive pulmonary disease: burden of disease and immune response. Am J Respir Crit Care Med 172: 195–199. 15805178 10.1164/rccm.200412-1747OC PMC2718466

[23] Sethi S, Evans N, Grant BJ, Murphy TF (2002) New strains of bacteria and exacerbations of chronic obstructive pulmonary disease. N Engl J Med 347: 465–471. 12181400 10.1056/NEJMoa012561

[24] Sethi S, Murphy TF (2001) Bacterial Infection in Chronic Obstructive Pulmonary Disease in 2000: a State-of-the-Art Review. Clin Microbiol Rev 14: 336–363. 11292642 10.1128/CMR.14.2.336-363.2001 PMC88978

[25] NIH-NHLBI website. Morbidity and Mortality: 2009 Chart Book on Cardiovascular, Lung, and Blood Diseases. Available: http://www.nhlbi.nih.gov/resources/docs/2009_ChartBook.pdf. Accessed 2013 June 4.

[26] Strassels SA, Smith DH, Sullivan SD, Mahajan PS (2001) The costs of treating COPD in the United States. Chest 119: 344–352. 11171708 10.1378/chest.119.2.344

[27] Sullivan SD, Ramsey SD, Lee TA (2000) The economic burden of COPD. Chest 117: 5S–9S. 10673466 10.1378/chest.117.2_suppl.5s

[28] Hunter MH, King DE (2001) COPD: management of acute exacerbations and chronic stable disease. Am Fam Physician 64: 603–612. 11529259

[29] Hurd S (2000) The impact of COPD on lung health worldwide: epidemiology and incidence. Chest 117: 1S–4S. 10673465 10.1378/chest.117.2_suppl.1s

[30] World Health Organization website. World Health Statistics 2008. Available: http://www.who.int/gho/publications/world_health_statistics/EN_WHS08_Full.pdf. Accessed 2013 June 4.

[31] World Health Organization website. Fact Sheet: The Top Ten Causes of Death (2008). Available: http://www.who.int/mediacentre/factsheets/fs310_2008.pdf.Accessed 2013 June 4.

[32] Ahmed A, Broides A, Givon-Lavi N, Peled N, Dagan R, et al (2008) Clinical and laboratory aspects of Moraxella catarrhalis bacteremia in children. Pediatr Infect Dis J 27: 459–461. 18360302 10.1097/INF.0b013e3181646d82

[33] Berner R, Schumacher RF, Brandis M, Forster J (1996) Colonization and infection with *Moraxella catarrhalis* in childhood. Eur J Clin Microbiol Infect Dis 15: 506–509. 8839647 10.1007/BF01691320

[34] Neumayer U, Schmidt HK, Mellwig KP, Kleikamp G (1999) Moraxella catarrhalis endocarditis: report of a case and literature review. J Heart Valve Dis 8: 114–117. 10096493

[35] Stefanou J, Agelopoulou AV, Sipsas NV, Smilakou N, Avlami A (2000) Moraxella catarrhalis endocarditis: case report and review of the literature. Scand J Infect Dis 32: 217–218. 10826914 10.1080/003655400750045394

[36] Thorsson B, Haraldsdottir V, Kristjansson M (1998) Moraxella catarrhalis bacteraemia. A report on 3 cases and a review of the literature. Scand J Infect Dis 30: 105–109. 9730292 10.1080/003655498750003447

[37] Turner HR, Taylor MR, Lockwood WR (1985) Branhamella catarrhalis endocarditis in a patient receiving hemodialysis. South Med J 78: 1021–1022. 4023776 10.1097/00007611-198508000-00041

[38] Utsunomiya T, Nakahara K, Kuramochi M, Hashiba K, Uzuka Y, et al (1984) [Branhamella (Neisseria) catarrhalis endocarditis after insertion of a mitral prosthesis: a case report]. Nippon Naika Gakkai Zasshi 73: 1506–1511. 6520515 10.2169/naika.73.1506

[39] Catlin BW (1990) *Branhamella catarrhalis*: an organism gaining respect as a pathogen. Clin Microbiol Rev 3: 293–320. 2121328 10.1128/cmr.3.4.293 PMC358165

[40] Christensen JJ (1999) *Moraxella (Branhamella) catarrhalis*: clinical, microbiological and immunological features in lower respiratory tract infections. APMIS Suppl 88: 1–36. 10189833

[41] Nigrovic LE, Kuppermann N, Malley R (2008) Children with bacterial meningitis presenting to the emergency department during the pneumococcal conjugate vaccine era. Acad Emerg Med 15: 522–528. 18616437 10.1111/j.1553-2712.2008.00117.x

[42] Brook I (2005) The role of bacteria in chronic rhinosinusitis. Otolaryngol Clin North Am 38: 1171–1192. 16326177 10.1016/j.otc.2005.08.007

[43] Brook I (2005) Microbiology and antimicrobial management of sinusitis. J Laryngol Otol 119: 251–258. 15949076 10.1258/0022215054020304

[44] Brook I, Foote PA, Frazier EH (2005) Microbiology of acute exacerbation of chronic sinusitis. Ann Otol Rhinol Laryngol 114: 573–576. 16134356 10.1177/000348940511400714

[45] Bingen E, Cohen R, Jourenkova N, Gehanno P (2005) Epidemiologic study of conjunctivitis-otitis syndrome. Pediatr Infect Dis J 24: 731–732. 16094231 10.1097/01.inf.0000172939.13159.3b

[46] Buznach N, Dagan R, Greenberg D (2005) Clinical and bacterial characteristics of acute bacterial conjunctivitis in children in the antibiotic resistance era. Pediatr Infect Dis J 24: 823–828. 16148850 10.1097/01.inf.0000178066.24569.98

[47] Jacobs MR, Bajaksouzian S, Windau A, Good CE, Lin G, et al (2004) Susceptibility of Streptococcus pneumoniae, Haemophilus influenzae, and Moraxella catarrhalis to 17 oral antimicrobial agents based on pharmacodynamic parameters: 1998–2001 U S Surveillance Study. Clin Lab Med 24: 503–530. 15177851 10.1016/j.cll.2004.03.008

[48] Klugman KP (1996) The clinical relevance of in-vitro resistance to penicillin, ampicillin, amoxycillin and alternative agents, for the treatment of community-acquired pneumonia caused by *Streptococcus pneumoniae, Haemophilus influenzae* and *Moraxella catarrhalis*. J Antimicrob Chemother 38 Suppl A: 133–140. 10.1093/jac/38.suppl_a.133 8858479

[49] Manninen R, Huovinen P, Nissinen A (1997) Increasing antimicrobial resistance in Streptococcus pneumoniae, Haemophilus influenzae and Moraxella catarrhalis in Finland. J Antimicrob Chemother 40: 387–392. 9338492 10.1093/jac/40.3.387

[50] Richter SS, Winokur PL, Brueggemann AB, Huynh HK, Rhomberg PR, et al (2000) Molecular characterization of the beta-lactamases from clinical isolates of Moraxella (Branhamella) catarrhalis obtained from 24 U.S. medical centers during 1994–1995 and 1997–1998. Antimicrob Agents Chemother 44: 444–446. 10639381 10.1128/aac.44.2.444-446.2000 PMC89702

[51] Kadry AA, Fouda SI, Elkhizzi NA, Shibl AM (2003) Correlation between susceptibility and BRO type enzyme of Moraxella catarrhalis strains. Int J Antimicrob Agents 22: 532–536. 14602374 10.1016/s0924-8579(03)00158-4

[52] Schmitz FJ, Beeck A, Perdikouli M, Boos M, Mayer S, et al (2002) Production of BRO beta-lactamases and resistance to complement in European Moraxella catarrhalis isolates. J Clin Microbiol 40: 1546–1548. 11923393 10.1128/JCM.40.4.1546-1548.2002 PMC140350

[53] Johnson DM, Sader HS, Fritsche TR, Biedenbach DJ, Jones RN (2003) Susceptibility trends of haemophilus influenzae and Moraxella catarrhalis against orally administered antimicrobial agents: five-year report from the SENTRY Antimicrobial Surveillance Program. Diagn Microbiol Infect Dis 47: 373–376. 12967753 10.1016/s0732-8893(03)00089-0

[54] Esel D, Ay-Altintop Y, Yagmur G, Gokahmetoglu S, Sumerkan B (2007) Evaluation of susceptibility patterns and BRO beta-lactamase types among clinical isolates of Moraxella catarrhalis. Clin Microbiol Infect 13: 1023–1025. 17608812 10.1111/j.1469-0691.2007.01776.x

[55] Bootsma HJ, Aerts PC, Posthuma G, Harmsen T, Verhoef J, et al (1999) *Moraxella (Branhamella) catarrhalis* BRO beta-lactamase: a lipoprotein of gram-positive origin? J Bacteriol 181: 5090–5093. 10438784 10.1128/jb.181.16.5090-5093.1999 PMC94001

[56] Bootsma HJ, van Dijk H, Vauterin P, Verhoef J, Mooi FR (2000) Genesis of BRO beta-lactamase-producing *Moraxella catarrhalis*: evidence for transformation-mediated horizontal transfer. Mol Microbiol 36: 93–104. 10760166 10.1046/j.1365-2958.2000.01828.x

[57] Garcia-Rodriguez JA, Fresnadillo Martinez MJ (2002) Dynamics of nasopharyngeal colonization by potential respiratory pathogens. J Antimicrob Chemother 50 Suppl S259–73. 10.1093/jac/dkf506 12556435

[58] Faden H, Harabuchi Y, Hong JJ (1994) Epidemiology of Moraxella catarrhalis in children during the first 2 years of life: relationship to otitis media. J Infect Dis 169: 1312–1317. 8195609 10.1093/infdis/169.6.1312

[59] Gerlach RG, Hensel M (2007) Protein secretion systems and adhesins: the molecular armory of Gram-negative pathogens. Int J Med Microbiol 297: 401–415. 17482513 10.1016/j.ijmm.2007.03.017

[60] Beachey EH (1981) Bacterial adherence: adhesin-receptor interactions mediating the attachment of bacteria to mucosal surface. J Infect Dis 143: 325–345. 7014727 10.1093/infdis/143.3.325

[61] Boyle EC, Finlay BB (2003) Bacterial pathogenesis: exploiting cellular adherence. Curr Opin Cell Biol 15: 633–639. 14519399 10.1016/s0955-0674(03)00099-1

[62] Hauck CR (2002) Cell adhesion receptors - signaling capacity and exploitation by bacterial pathogens. Med Microbiol Immunol 191: 55–62. 12410343 10.1007/s00430-002-0119-0

[63] Jacob-Dubuisson F, Locht C, Antoine R (2001) Two-partner secretion in Gram-negative bacteria: a thrifty, specific pathway for large virulence proteins. Mol Microbiol 40: 306–313. 11309114 10.1046/j.1365-2958.2001.02278.x

[64] Niemann HH, Schubert WD, Heinz DW (2004) Adhesins and invasins of pathogenic bacteria: a structural view. Microbes Infect 6: 101–112. 14738899 10.1016/j.micinf.2003.11.001

[65] St Geme JW 3rd (1997) Bacterial adhesins: determinants of microbial colonization and pathogenicity. Adv Pediatr 44: 43–72. 9265967

[66] Lafontaine ER, Cope LD, Aebi C, Latimer JL, McCracken GH Jr, et al (2000) The UspA1 protein and a second type of UspA2 protein mediate adherence of *Moraxella catarrhalis* to human epithelial cells in vitro. J Bacteriol 182: 1364–1373. 10671460 10.1128/jb.182.5.1364-1373.2000 PMC94425

[67] Balder R, Hassel J, Lipski S, Lafontaine ER (2007) Moraxella catarrhalis strain O35E expresses two filamentous hemagglutinin-like proteins that mediate adherence to human epithelial cells. Infect Immun 75: 2765–2775. 17371858 10.1128/IAI.00079-07 PMC1932885

[68] Plamondon P, Luke NR, Campagnari AA (2007) Identification of a novel two-partner secretion locus in Moraxella catarrhalis. Infect Immun 75: 2929–2936. 17420235 10.1128/IAI.00396-07 PMC1932880

[69] Balder R, Krunkosky TM, Nguyen CQ, Feezel L, Lafontaine ER (2009) Hag mediates adherence of Moraxella catarrhalis to ciliated human airway cells. Infect Immun 77: 4597–4608. 19667048 10.1128/IAI.00212-09 PMC2747927

[70] Bullard B, Lipski SL, Lafontaine ER (2005) Hag directly mediates the adherence of Moraxella catarrhalis to human middle ear cells. Infect Immun 73: 5127–5136. 16041029 10.1128/IAI.73.8.5127-5136.2005 PMC1201204

[71] Akimana C, Lafontaine ER (2007) The Moraxella catarrhalis outer membrane protein CD contains two distinct domains specifying adherence to human lung cells. FEMS Microbiol Lett 271: 12–19. 17391370 10.1111/j.1574-6968.2007.00693.x

[72] Holm MM, Vanlerberg SL, Foley IM, Sledjeski DD, Lafontaine ER (2004) The Moraxella catarrhalis porin-like outer membrane protein CD is an adhesin for human lung cells. Infect Immun 72: 1906–1913. 15039309 10.1128/IAI.72.4.1906-1913.2004 PMC375153

[73] Lipski SL, Akimana C, Timpe JM, Wooten RM, Lafontaine ER (2007) The Moraxella catarrhalis Autotransporter McaP Is a Conserved Surface Protein That Mediates Adherence to Human Epithelial Cells through Its N-Terminal Passenger Domain. Infect Immun 75: 314–324. 17088358 10.1128/IAI.01330-06 PMC1828417

[74] Timpe JM, Holm MM, Vanlerberg SL, Basrur V, Lafontaine ER (2003) Identification of a Moraxella catarrhalis outer membrane protein exhibiting both adhesin and lipolytic activities. Infect Immun 71: 4341–4350. 12874311 10.1128/IAI.71.8.4341-4350.2003 PMC166007

[75] Sambrook J, Russell DW (2001) Molecular Cloning: A Laboratory Manual (Third Edition): Cold Spring Harbor Laboratory Press.

[76] Murphy TF, Loeb MR (1989) Isolation of the outer membrane of *Branhamella catarrhalis* . Microb Pathog 6: 159–174. 2500575 10.1016/0882-4010(89)90066-1

[77] Cope LD, Lafontaine ER, Slaughter CA, Hasemann CA Jr, Aebi C, et al (1999) Characterization of the *Moraxella catarrhalis uspA1* and *uspA2* genes and their encoded products. J Bacteriol 181: 4026–4034. 10383971 10.1128/jb.181.13.4026-4034.1999 PMC93893

[78] Patrick CC, Kimura A, Jackson MA, Hermanstorfer L, Hood A, et al (1987) Antigenic characterization of the oligosaccharide portion of the lipooligosaccharide of nontypable *Haemophilus influenzae* . Infect Immun 55: 2902–2911. 2445682 10.1128/iai.55.12.2902-2911.1987 PMC260004

[79] Song JM, Hossain J, Yoo DG, Lipatov AS, Davis CT, et al (2010) Protective immunity against H5N1 influenza virus by a single dose vaccination with virus-like particles. Virology 405: 165–175. 20580392 10.1016/j.virol.2010.05.034 PMC2925114

[80] Helminen ME, Maciver I, Latimer JL, Cope LD, McCracken GH Jr, et al (1993) A major outer membrane protein of *Moraxella catarrhalis* is a target for antibodies that enhance pulmonary clearance of the pathogen in an animal model. Infect Immun 61: 2003–2010. 7683000 10.1128/iai.61.5.2003-2010.1993 PMC280795

[81] Pearson MM, Lafontaine ER, Wagner NJ, St Geme JW 3rd, Hansen EJ (2002) A hag mutant of Moraxella catarrhalis strain O35E is deficient in hemagglutination, autoagglutination, and immunoglobulin D-binding activities. Infect Immun 70: 4523–4533. 12117964 10.1128/IAI.70.8.4523-4533.2002 PMC128162

[82] Aebi C, Lafontaine ER, Cope LD, Latimer JL, Lumbley SL, et al (1998) Phenotypic effect of isogenic *uspA1* and *uspA2* mutations on *Moraxella catarrhalis* 035E. Infect Immun 66: 3113–3119. 9632574 10.1128/iai.66.7.3113-3119.1998 PMC108321

[83] Murphy TF, Kirkham C, DeNardin E, Sethi S (1999) Analysis of antigenic structure and human immune response to outer membrane protein CD of *Moraxella catarrhalis* . Infect Immun 67: 4578–4585. 10456903 10.1128/iai.67.9.4578-4585.1999 PMC96781

[84] Luke NR, Jurcisek JA, Bakaletz LO, Campagnari AA (2007) Contribution of Moraxella catarrhalis type IV pili to nasopharyngeal colonization and biofilm formation. Infect Immun 75: 5559–5564. 17908808 10.1128/IAI.00946-07 PMC2168369

[85] Bakaletz LO, Murwin DM, Billy JM (1995) Adenovirus serotype 1 does not act synergistically with *Moraxella (Branhamella) catarrhalis* to induce otitis media in the chinchilla. Infect Immun 63: 4188–4190. 7558341 10.1128/iai.63.10.4188-4190.1995 PMC173592

[86] Hoopman TC, Liu W, Joslin SN, Pybus C, Sedillo JL, et al (2012) Use of the chinchilla model for nasopharyngeal colonization to study gene expression by Moraxella catarrhalis. Infect Immun 80: 982–995. 22184412 10.1128/IAI.05918-11 PMC3294655

[87] Murphy TF, Kirkham C, Liu DF, Sethi S (2003) Human immune response to outer membrane protein CD of Moraxella catarrhalis in adults with chronic obstructive pulmonary disease. Infect Immun 71: 1288–1294. 12595444 10.1128/IAI.71.3.1288-1294.2003 PMC148877

[88] LaFontaine ER, Snipes LE, Bullard B, Brauer AL, Sethi S, et al (2009) Identification of domains of the Hag/MID surface protein recognized by systemic and mucosal antibodies in adults with chronic obstructive pulmonary disease following clearance of Moraxella catarrhalis. Clin Vaccine Immunol 16: 653–659. 19321697 10.1128/CVI.00460-08 PMC2681595

[89] Murphy TF, Brauer AL, Aebi C, Sethi S (2005) Antigenic specificity of the mucosal antibody response to Moraxella catarrhalis in chronic obstructive pulmonary disease. Infect Immun 73: 8161–8166. 16299311 10.1128/IAI.73.12.8161-8166.2005 PMC1307080

[90] Murphy TF, Brauer AL, Aebi C, Sethi S (2005) Identification of surface antigens of Moraxella catarrhalis as targets of human serum antibody responses in chronic obstructive pulmonary disease. Infect Immun 73: 3471–3478. 15908376 10.1128/IAI.73.6.3471-3478.2005 PMC1111810

[91] Stutzmann Meier P, Heiniger N, Troller R, Aebi C (2003) Salivary antibodies directed against outer membrane proteins of Moraxella catarrhalis in healthy adults. Infect Immun 71: 6793–6798. 14638765 10.1128/IAI.71.12.6793-6798.2003 PMC308912

[92] Meier PS, Freiburghaus S, Martin A, Heiniger N, Troller R, et al (2003) Mucosal immune response to specific outer membrane proteins of Moraxella catarrhalis in young children. Pediatr Infect Dis J 22: 256–262. 12634588 10.1097/01.inf.0000054827.86683.bd

[93] Chen D, Barniak V, VanDerMeid KR, McMichael JC (1999) The levels and bactericidal capacity of antibodies directed against the UspA1 and UspA2 outer membrane proteins of *Moraxella (Branhamella) catarrhalis* in adults and children. Infect Immun 67: 1310–1316. 10024576 10.1128/iai.67.3.1310-1316.1999 PMC96462

[94] Mathers K, Leinonen M, Goldblatt D (1999) Antibody response to outer membrane proteins of *Moraxella catarrhalis* in children with otitis media. Pediatr Infect Dis J 18: 982–988. 10571435 10.1097/00006454-199911000-00010

[95] Eskola J, Kilpi T, Palmu A, Jokinen J, Haapakoski J, et al (2001) Efficacy of a pneumococcal conjugate vaccine against acute otitis media. N Engl J Med 344: 403–409. 11172176 10.1056/NEJM200102083440602

[96] Prymula R, Peeters P, Chrobok V, Kriz P, Novakova E, et al (2006) Pneumococcal capsular polysaccharides conjugated to protein D for prevention of acute otitis media caused by both Streptococcus pneumoniae and non-typable Haemophilus influenzae: a randomised double-blind efficacy study. Lancet 367: 740–748. 16517274 10.1016/S0140-6736(06)68304-9

[97] Revai K, McCormick DP, Patel J, Grady JJ, Saeed K, et al (2006) Effect of pneumococcal conjugate vaccine on nasopharyngeal bacterial colonization during acute otitis media. Pediatrics 117: 1823–1829. 16651345 10.1542/peds.2005-1983

[98] Brook I, Gober AE (2007) Frequency of recovery of pathogens from the nasopharynx of children with acute maxillary sinusitis before and after the introduction of vaccination with the 7-valent pneumococcal vaccine. Int J Pediatr Otorhinolaryngol 71: 575–579. 17267051 10.1016/j.ijporl.2006.10.025

[99] Brook I, Foote PA, Hausfeld JN (2006) Frequency of recovery of pathogens causing acute maxillary sinusitis in adults before and after introduction of vaccination of children with the 7-valent pneumococcal vaccine. J Med Microbiol 55: 943–946. 16772423 10.1099/jmm.0.46346-0

[100] Becker PD, Bertot GM, Souss D, Ebensen T, Guzman CA, et al (2007) Intranasal vaccination with recombinant outer membrane protein CD and adamantylamide dipeptide as the mucosal adjuvant enhances pulmonary clearance of Moraxella catarrhalis in an experimental murine model. Infect Immun 75: 1778–1784. 17101651 10.1128/IAI.01081-06 PMC1865668

[101] Liu DF, McMichael JC, Baker SM (2007) Moraxella catarrhalis outer membrane protein CD elicits antibodies that inhibit CD binding to human mucin and enhance pulmonary clearance of M. catarrhalis in a mouse model. Infect Immun 75: 2818–2825. 17403868 10.1128/IAI.00074-07 PMC1932855

[102] Peng D, Choudhury BP, Petralia RS, Carlson RW, Gu XX (2005) Roles of 3-deoxy-D-manno-2-octulosonic acid transferase from Moraxella catarrhalis in lipooligosaccharide biosynthesis and virulence. Infect Immun 73: 4222–4230. 15972513 10.1128/IAI.73.7.4222-4230.2005 PMC1168618

[103] Maciver I, Unhanand M, McCracken GH Jr, Hansen EJ (1993) Effect of immunization of pulmonary clearance of *Moraxella catarrhalis* in an animal model. J Infect Dis 168: 469–472. 8335988 10.1093/infdis/168.2.469

[104] Unhanand M, Maciver I, Ramilo O, Arencibia-Mireles O, Argyle JC, et al (1992) Pulmonary clearance of *Moraxella catarrhalis* in an animal model. J Infect Dis 165: 644–650. 1532405 10.1093/infdis/165.4.644

[105] Kyd JM, Cripps AW, Murphy TF (1998) Outer-membrane antigen expression by *Moraxella (Branhamella) catarrhalis* influences pulmonary clearance. J Med Microbiol 47: 159–168. 9879959 10.1099/00222615-47-2-159

[106] Murphy TF, Kyd JM, John A, Kirkham C, Cripps AW (1998) Enhancement of pulmonary clearance of *Moraxella (Branhamella) catarrhalis* following immunization with outer membrane protein CD in a mouse model. J Infect Dis 178: 1667–1675. 9815219 10.1086/314501

[107] Armbruster CE, Hong W, Pang B, Weimer KE, Juneau RA, et al.. (2010) Indirect Pathogenicity of Haemophilus influenzae and Moraxella catarrhalis in Polymicrobial Otitis Media Occurs via Interspecies Quorum Signaling. MBio 1. 10.1128/mBio.00102-10 PMC2925075 20802829

[108] Brockson ME, Novotny LA, Jurcisek JA, McGillivary G, Bowers MR, et al (2012) Respiratory syncytial virus promotes Moraxella catarrhalis-induced ascending experimental otitis media. PLoS One 7: e40088. 22768228 10.1371/journal.pone.0040088 PMC3387005

[109] Bakaletz LO (2009) Chinchilla as a robust, reproducible and polymicrobial model of otitis media and its prevention. Expert Rev Vaccines 8: 1063–1082. 19627188 10.1586/erv.09.63

[110] Forsgren A, Riesbeck K, Janson H (2008) Protein D of Haemophilus influenzae: a protective nontypeable H. influenzae antigen and a carrier for pneumococcal conjugate vaccines. Clin Infect Dis 46: 726–731. 18230042 10.1086/527396

[111] Giebink GS (1999) Otitis media: the chinchilla model. Microb Drug Resist 5: 57–72. 10332723 10.1089/mdr.1999.5.57

[112] Giebink GS (1997) Vaccination against middle-ear bacterial and viral pathogens. Ann N Y Acad Sci 830: 330–352. 9616694 10.1111/j.1749-6632.1997.tb51906.x

[113] Bakaletz LO, Daniels RL, Lim DJ (1993) Modeling adenovirus type 1-induced otitis media in the chinchilla: effect on ciliary activity and fluid transport function of eustachian tube mucosal epithelium. J Infect Dis 168: 865–872. 8397268 10.1093/infdis/168.4.865

[114] Suzuki K, Bakaletz LO (1994) Synergistic effect of adenovirus type 1 and nontypeable Haemophilus influenzae in a chinchilla model of experimental otitis media. Infect Immun 62: 1710–1718. 8168932 10.1128/iai.62.5.1710-1718.1994 PMC186390

[115] Bakaletz LO (1995) Viral potentiation of bacterial superinfection of the respiratory tract. Trends Microbiol 3: 110–114. 7773588 10.1016/s0966-842x(00)88892-7

[116] Miyamoto N, Bakaletz LO (1997) Kinetics of the ascension of NTHi from the nasopharynx to the middle ear coincident with adenovirus-induced compromise in the chinchilla. Microb Pathog 23: 119–126. 9245624 10.1006/mpat.1997.0140

[117] Kennedy BJ, Novotny LA, Jurcisek JA, Lobet Y, Bakaletz LO (2000) Passive transfer of antiserum specific for immunogens derived from a nontypeable Haemophilus influenzae adhesin and lipoprotein D prevents otitis media after heterologous challenge. Infect Immun 68: 2756–2765. 10768970 10.1128/iai.68.5.2756-2765.2000 PMC97485

[118] Bakaletz LO, Kennedy BJ, Novotny LA, Duquesne G, Cohen J, et al (1999) Protection against development of otitis media induced by nontypeable Haemophilus influenzae by both active and passive immunization in a chinchilla model of virus-bacterium superinfection. Infect Immun 67: 2746–2762. 10338477 10.1128/iai.67.6.2746-2762.1999 PMC96578

[119] Novotny LA, Jurcisek JA, Pichichero ME, Bakaletz LO (2000) Epitope mapping of the outer membrane protein P5-homologous fimbrin adhesin of nontypeable Haemophilus influenzae. Infect Immun 68: 2119–2128. 10722609 10.1128/iai.68.4.2119-2128.2000 PMC97393

[120] McGillivary G, Bakaletz LO (2010) The multifunctional host defense peptide SPLUNC1 is critical for homeostasis of the mammalian upper airway. PLoS One 5: e13224. 20949060 10.1371/journal.pone.0013224 PMC2951362

[121] McGillivary G, Mason KM, Jurcisek JA, Peeples ME, Bakaletz LO (2009) Respiratory syncytial virus-induced dysregulation of expression of a mucosal beta-defensin augments colonization of the upper airway by non-typeable Haemophilus influenzae. Cell Microbiol 11: 1399–1408. 19500108 10.1111/j.1462-5822.2009.01339.x PMC2741182

[122] McGillivary G, Ray WC, Bevins CL, Munson RS Jr, Bakaletz LO (2007) A member of the cathelicidin family of antimicrobial peptides is produced in the upper airway of the chinchilla and its mRNA expression is altered by common viral and bacterial co-pathogens of otitis media. Mol Immunol 44: 2446–2458. 17113647 10.1016/j.molimm.2006.10.008 PMC1817667

[123] Harris RH, Wilk D, Bevins CL, Munson RS Jr, Bakaletz LO (2004) Identification and characterization of a mucosal antimicrobial peptide expressed by the chinchilla (Chinchilla lanigera) airway. J Biol Chem 279: 20250–20256. 14996845 10.1074/jbc.M400499200

[124] Kerschner JE, Khampang P, Samuels T (2010) Extending the chinchilla middle ear epithelial model for mucin gene investigation. Int J Pediatr Otorhinolaryngol 74: 980–985. 20591507 10.1016/j.ijporl.2010.05.009 PMC2922454

[125] Kerschner JE, Erdos G, Hu FZ, Burrows A, Cioffi J, et al (2010) Partial characterization of normal and Haemophilus influenzae-infected mucosal complementary DNA libraries in chinchilla middle ear mucosa. Ann Otol Rhinol Laryngol 119: 270–278. 20433028 10.1177/000348941011900411 PMC2910914

[126] Kerschner JE, Horsey E, Ahmed A, Erbe C, Khampang P, et al (2009) Gene expression differences in infected and noninfected middle ear complementary DNA libraries. Arch Otolaryngol Head Neck Surg 135: 33–39. 19153305 10.1001/archoto.2008.513 PMC2912141

[127] Meier PS, Troller R, Heiniger N, Hays JP, van Belkum A, et al (2006) Unveiling electrotransformation of Moraxella catarrhalis as a process of natural transformation. FEMS Microbiol Lett 262: 72–76. 16907741 10.1111/j.1574-6968.2006.00365.x

[128] Brooks MJ, Sedillo JL, Wagner N, Wang W, Attia AS, et al (2008) Moraxella catarrhalis binding to host cellular receptors is mediated by sequence-specific determinants not conserved among all UspA1 protein variants. Infect Immun 76: 5322–5329. 18678656 10.1128/IAI.00572-08 PMC2573313

[129] Hill DJ, Virji M (2003) A novel cell-binding mechanism of *Moraxella catarrhalis* ubiquitous surface protein UspA: specific targeting of the N-domain of carcinoembryonic antigen-related cell adhesion molecules by UspA1. Mol Microbiol 48: 117–129. 12657049 10.1046/j.1365-2958.2003.03433.x

[130] Hill DJ, Whittles C, Virji M (2012) A novel group of Moraxella catarrhalis UspA proteins mediates cellular adhesion via CEACAMs and vitronectin. PLoS One 7: e45452. 23049802 10.1371/journal.pone.0045452 PMC3458076

[131] Bookwalter JE, Jurcisek JA, Gray-Owen SD, Fernandez S, McGillivary G, et al (2008) A carcinoembryonic antigen-related cell adhesion molecule 1 homologue plays a pivotal role in nontypeable Haemophilus influenzae colonization of the chinchilla nasopharynx via the outer membrane protein P5-homologous adhesin. Infect Immun 76: 48–55. 17938212 10.1128/IAI.00980-07 PMC2223670

[132] Holm MM, Vanlerberg SL, Sledjeski DD, Lafontaine ER (2003) The Hag protein of Moraxella catarrhalis strain O35E is associated with adherence to human lung and middle ear cells. Infect Immun 71: 4977–4984. 12933840 10.1128/IAI.71.9.4977-4984.2003 PMC187358

[133] Aebi C, Lafontaine ER, Cope LD, Latimer JL, Lumbley SL, et al (1998) Phenotypic effect of isogenic uspA1 and uspA2 mutations on Moraxella catarrhalis 035E. Infect Immun 66: 3113–3119. 9632574 10.1128/iai.66.7.3113-3119.1998 PMC108321

